# Investigation of Mechanical and Hydrologic Characteristics of Porous Asphalt Pavement with a Geocell Composite

**DOI:** 10.3390/ma14123165

**Published:** 2021-06-09

**Authors:** Jaehun Ahn, Tan Hung Nguyen, In Kyoon Yoo, Jeongho Oh

**Affiliations:** 1 Department of Civil and Environmental Engineering, Pusan National University, Busan 46241, Korea; jahn@pusan.ac.kr; 2 Faculty of Architectural, Civil and Environmental Engineering, Nam Can Tho University, Can Tho 900000, Vietnam; nthung010189@gmail.com; 3 Department of Infrastructure Safety Research, Korea Institute of Civil Engineering and Building Technology, Goyang-si 10223, Korea; ikyoo@kict.re.kr; 4 Department of Railroad Infrastructure System Engineering, Korea National University of Transportation, Uiwang-si 16106, Korea

**Keywords:** geocell composite, porous asphalt pavement, hydrologic characteristics, falling weight deflectometer, modulus of elasticity

## Abstract

Porous asphalt pavement is a part of the permeable pavement system, which can be used to mitigate the negative impacts of urbanisation on the water hydrological cycle and environment. This study aims to assess the mechanical and hydrologic characteristics of porous asphalt pavements, with and without geocell composites, using a plate load test, falling weight deflectometer test, and rainfall simulation test. The corresponding results indicate that the elastic modulus of the unreinforced pavement is lower than that of the reinforced pavement. The analysis demonstrates that the use of geocell composites effectively increases the load-bearing capacity of the pavement. When the base layer is reinforced with geocells, its load-bearing capacity increases. Observation of the rainfall simulation tests on the reinforced pavement indicates that the reinforced pavement effectively handles the surface runoff.

## 1. Introduction

The permeable pavement system is a low impact development technology that can be used to mitigate the negative impacts of urbanisation on the water hydrological cycle. Permeable pavement systems have recently seen increasing use worldwide as an effective solution for controlling rainwater quantity and quality [1,2]. The high porosity of a permeable pavement system allows the infiltration of rainwater. This process allows for the filtering of pollutants associated with the rainwater. Therefore, permeable pavement systems can mitigate surface flooding and environmental pollution in urban areas [3]. The major design consideration of a permeable pavement system is its structure, which should satisfy the required mechanical and hydrologic characteristics [4]. However, the mechanical characteristics of permeable pavement systems are worse than those of conventional pavements owing to their high porosity [5]. To improve the mechanical characteristics of permeable pavement systems, geosynthetics have been used for reinforcement.

Geosynthetics have been used widely for the underlying layers of civil engineering constructions, such as slopes, retaining walls, foundations, and pavements, to improve their mechanical characteristics. To date, geosynthetics, including geogrids, geotextiles, geocells, geomembranes, etc., have been introduced into the market for engineers. Depending on the construction and purpose, each type of geosynthetic is considered for use. Geosynthetics have been installed in the subgrade or base/subbase layers of pavement systems to improve their mechanical characteristics [6]. The implementation of geosynthetics in pavement systems provides several benefits: (1) increasing the load-bearing capacity of the system, (2) increasing the resistance to permanent deformation of the pavement layers, and (3) increasing the resistance to fatigue of the asphalt layer [7]. Among the various geosynthetics, geocells provide the best base/subbase reinforcement to the pavement system under cyclic loading [7,8]. The cells are produced by high-density polyethylene or a novel polymer alloy. In addition, they are lightweight and easy to install on-site [9]. For on-site installation, the subgrade, base, or subbase is covered by a geocell layer; this honeycomb-structured formwork layer is filled with a material, such as sand, soil, aggregate, recycled asphalt, or concrete. 

Several studies have been conducted to examine the mechanical characteristics of pavements reinforced with geocells and geocell composites. Khalaj et al. (2015) experimented on unreinforced and reinforced pavements on-site using cyclic plate loading tests (PLTs) [10]. In their research, PLTs were conducted on a test pit with dimensions of 2 m × 2 m at a depth of 0.7 m. Four geocell layers were installed within a depth of 0.7 m, each with a thickness of 0.1 m. The vertical spacing of each layer was 0.06 m. The results showed that using a geocell to reinforce the pavement layers improved their mechanical characteristics. For the reinforced pavement, the permanent strain decreased from 53% to 63% compared to the unreinforced pavement. This study found that geocells help to distribute more stress horizontally than vertically. Another study by George et al. (2019) investigated the efficiency of using geocells to reinforce pavements [11]; their experiments were conducted in a large-scale laboratory, using repeated load box tests. The effect of using a geocell on the mechanical characteristics was evaluated on the basis of the resilient modulus and permanent deformation. Two different geocell heights of 0.1 m and 0.15 m were used for the investigation. In the test layer, a geocell layer was placed on a geosynthetic membrane and filled with reclaimed asphalt pavement. The reclaimed asphalt pavement was constructed in three layers, each compacted using a compactor. The results showed that the reinforced pavement has a resilient modulus of approximately 2.5–3.3 times more than the unreinforced pavement. Furthermore, the use of a geocell could reduce the permanent deformation of the pavement by 70% to 80%. The conclusion was made that using a geocell can help to reduce the pavement thickness, especially for the base layer. Saha and Mandal (2018) investigated the effect of using bamboo geocells to improve the mechanical characteristics of recycled asphalt pavement [12]. In this research, recycled asphalt pavement was constructed inside the steel box and comprised three layers: surface, base, and subgrade. The surface and base layers were constructed from reclaimed asphalt pavement and the base was reinforced with bamboo geocells. The subgrade layer comprised soft marine clay. The bamboo geocells were manufactured using 0.01 m wide bamboo strips, 0.2 m in diameter, and 0.1 m in height. The results showed that when geocells were used, the load-bearing capacity of the pavement could increase by 63.3%. Moreover, the combination of geocells and a geogrid fabricated from bamboo could increase the load-bearing capacity of the pavement by 80%.

Studies have shown that the use of a geocell for reinforcing pavements could improve their mechanical characteristics. However, there is a gap in the research regarding its effects on the mechanical and hydrologic characteristics of a porous asphalt pavement. This study aims to assess the effect of using a geocell composite on the mechanical and hydrologic characteristics of a porous asphalt pavement. Two pavements, with and without geocell composite reinforcement, were evaluated using a PLT, the falling weight deflectometer (FWD) test, and rainfall simulation test. On the basis of the test results, the elastic modulus values were back-calculated and the infiltration rates of water flowing through the pavement body were evaluated. 

## 2. Porous Asphalt Pavement, Test Equipment, and Program

### 2.1. Porous Asphalt Pavement

**Figure 1:**
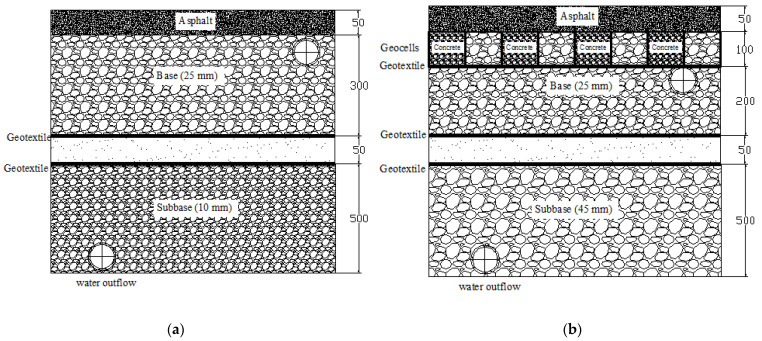
Porous asphalt pavements: (**a**) unreinforced; (**b**) reinforced with geocell composite.

In this study, two pavements, both with a thickness of 900 mm, were investigated. One was a porous asphalt pavement without a geocell composite (unreinforced pavement) and the other a porous asphalt pavement with a geocell composite at the base (reinforced pavement); they were constructed with dimensions of 2.3 m × 10.9 m, with structural layers as the surface, base, subbase, and subgrade. Figure 1 shows the structure of the two pavements.

**Figure 2:**
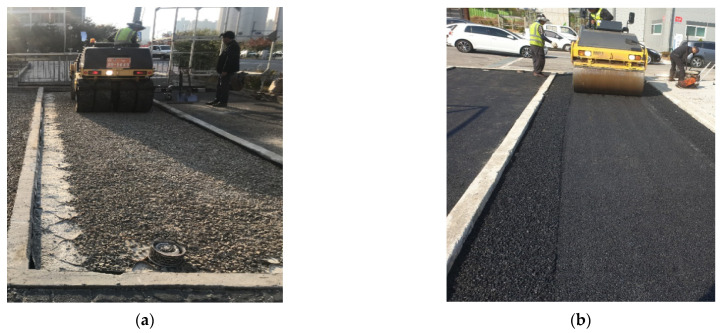
Compact asphalt pavement layer: (**a**) base layer; (**b**) asphalt layer.

The surfaces of the two pavements in this study were both constructed from a porous asphalt mixture, with a thickness of 50 mm. The porous asphalt mixtures were designed with an open-graded aggregate, with a nominal maximum aggregate size of 10 mm and a binder of PG76-22. A roller compactor (Kanto Tekko Co., Ltd., Koga City, Ibaraki, Japan) was used to compact these mixtures to fabricate the surface with a target porosity of 20%. Figure 2 shows the porous asphalt being compacted.

**Figure 3:**
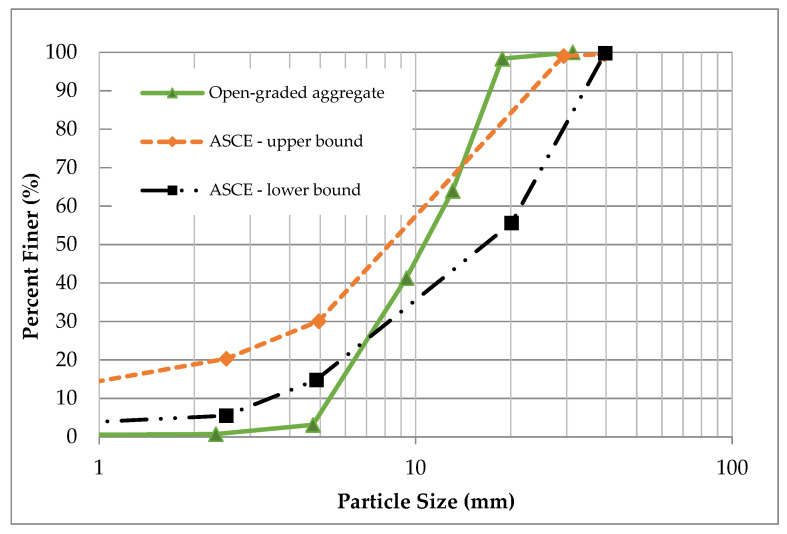
Aggregate gradation of the base layer.

For the base, an open-graded aggregate material was used whose gradation adhered to the guide for permeable bases in the American Society of Civil Engineers Manual [13]. The aggregate gradation is shown in Figure 3. 

**Figure 4:**
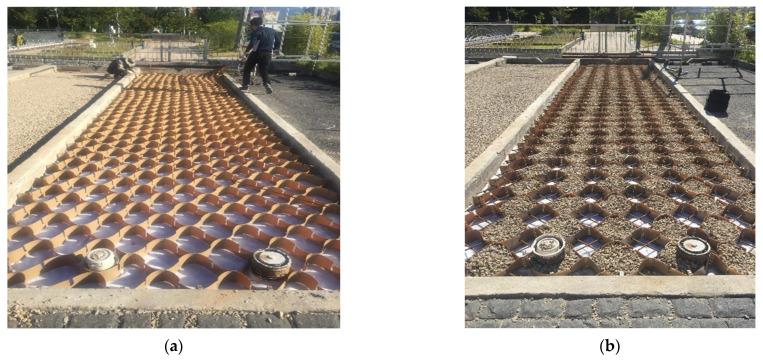
Laying and filling the geocells: (**a**) laying the geocells; (**b**) filling the geocells with aggregate.

The geocell composites in this study comprised geocells and steel bars. The geocell was fabricated from high-density polyethylene (HDPE), with a height of 100 mm, a width of 300 mm, and a sheet thickness of 0.8 mm. To reduce the deformation of the geocells, steel bars with a diameter of 10 mm were used as a reinforcement. The first row of geocells was filled with concrete, with a compressive strength of 21 MPa, while the second row was filled with an open-graded material. By this way, the porous asphalt pavement not only allowed for water to infiltrate through the base layer, but also obtained a high mechanical performance. Figure 4 shows the geocell layer after filling with concrete and the open-graded aggregate material.

**Figure 5:**
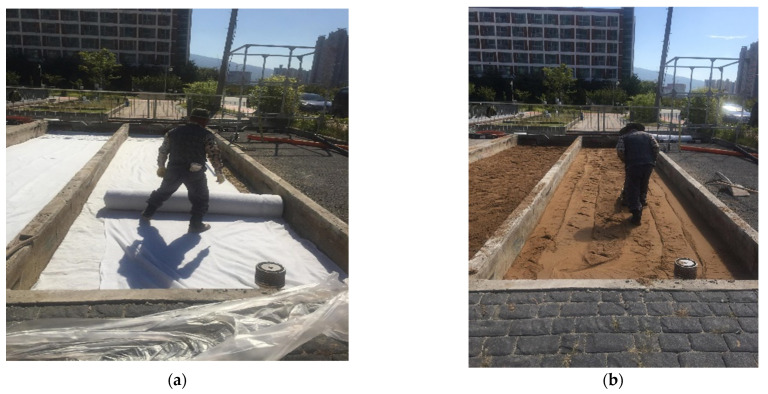
Laying the geotextile layer and compacting the sand layer (**a**) laying the geotextile layer; (**b**) vibratory compaction.

For the subbase, an aggregate material with a nominal maximum aggregate size of 10 mm and 45 mm was used for the unreinforced and reinforced pavement, respectively. The subgrade of the two pavements comprised a vibration-compacted sand layer, as shown in Figure 5. 

### 2.2. Test Equipment

#### 2.2.1. Plate Load Test

**Figure 6:**
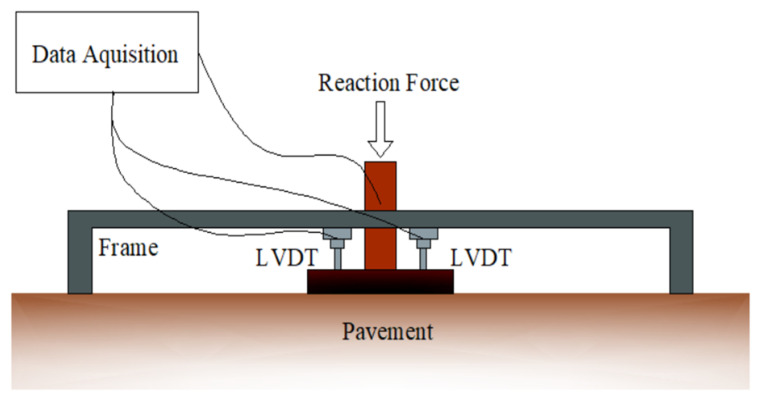
Schematic for PLT setup.

**Figure 7:**
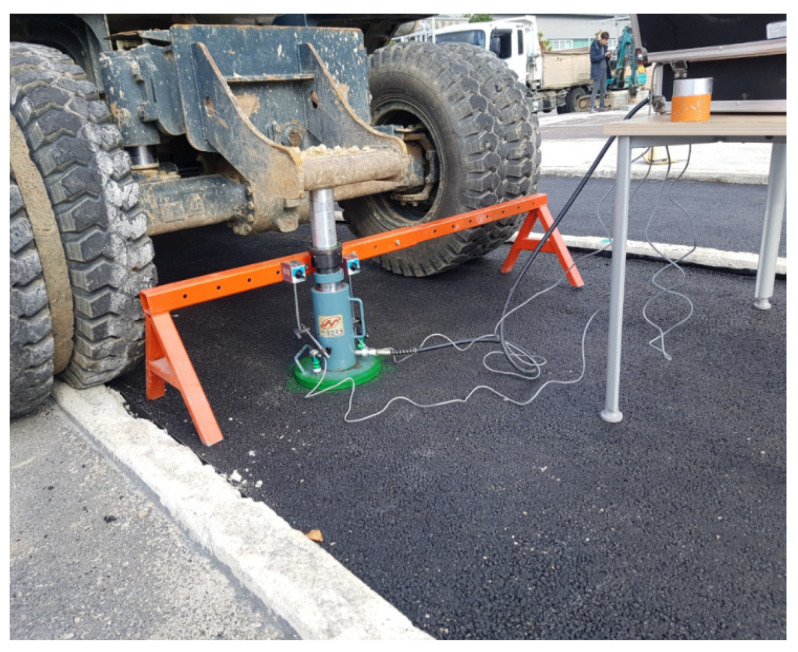
PLT setup for the pavements.

A steel rigid circular plate with a diameter of 300 mm was used as the loading surface for the PLT in this study. The normal stress on the plate was distributed by the reaction force from the excavator. The value of the force was controlled by a hand-operated hydraulic jack. Two linear variable differential transformers (LVDTs) were placed on the plate to measure the deformation of the pavements. Data, including force and deformations, were automatically recorded by the data acquisition system. A schematic of the test setup is shown in Figure 6. The test setup for the pavement is displayed in Figure 7.

#### 2.2.2. Falling Weight Deflectometer Test

**Figure 8:**
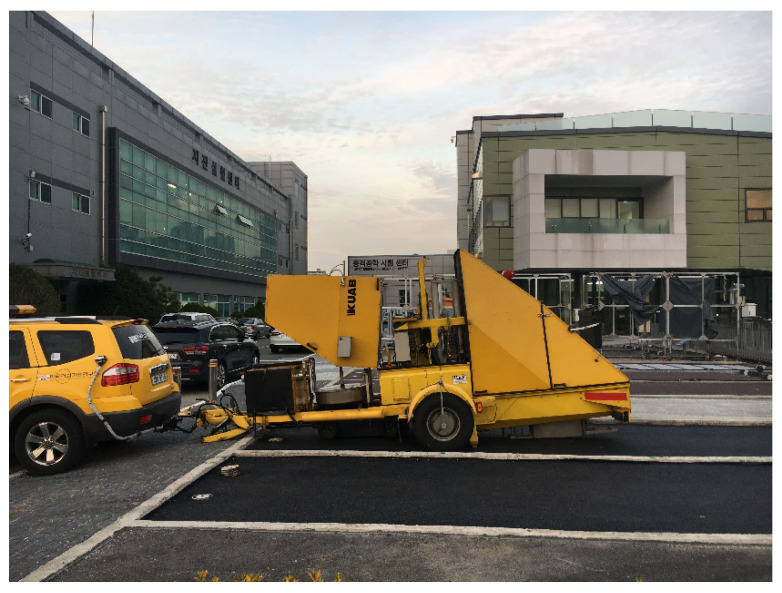
FWD test for pavements.

**Figure 9:**
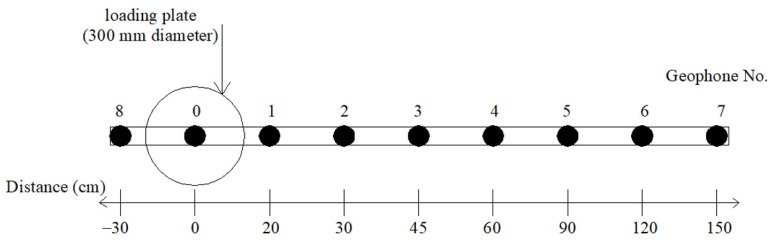
FWD geophone position.

Falling weight deflectometer (FWD) tests were conducted on the two pavements to evaluate the layer modulus, as illustrated in Figure 8. The equipment is a trailer-mounted device that measures the surface deflections of a 4535 kgf weight using geophones, as shown in Figure 9. In addition, air and pavement temperatures were recorded during the FWD data collection to take into account the effect of temperature on the measured deflection basins, which can be utilised to back-calculate the layer modulus.

**Figure 10:**
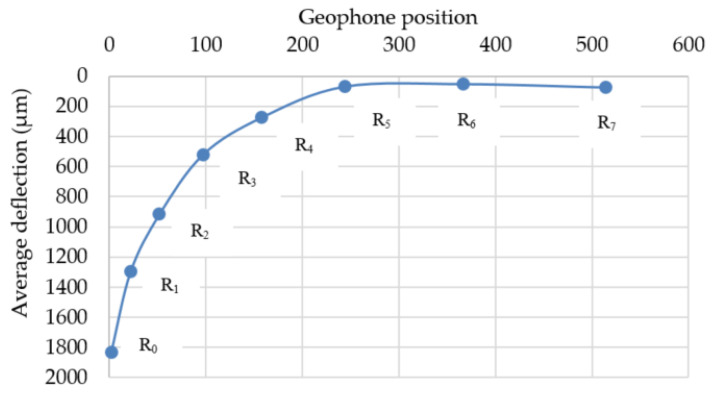
Example of FWD deflection basins’ layout.

For data collection in the FWD test, a total of nine geophones were installed for the two pavements, marked 0–7, as shown in Figure 9. The target load was 4535 kgf. The measurement from geophone no. 8 was only used to confirm the adequacy of the load transference. Figure 10 presents the average FWD deflection basins and geophone position. The deflections measured from geophone no. 0–1 generally represent the structural adequacy of the surface, 2–4 represent the structural adequacy of the middle layer structural adequacy, and 5–7 represent the structural adequacy of the subbase. An example of the FWD deflection basins’ layout is shown in Figure 10.

#### 2.2.3. Rainfall Simulation Test

**Figure 11:**
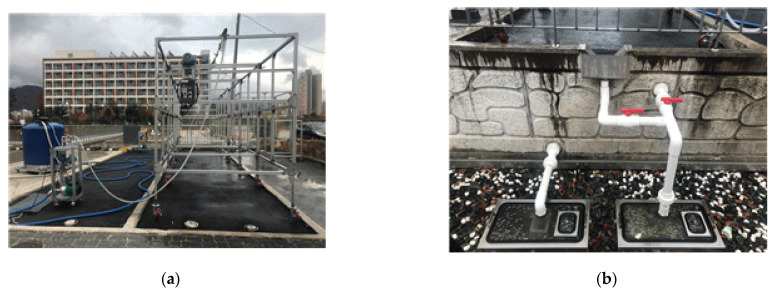
Rainfall simulation test (**a**) test setup; (**b**) outflow gauges for overflowing and draining.

Rainfall simulation tests were conducted to investigate the hydrologic characteristics of the porous asphalt pavement reinforced with geocell composites. In the experiment, rainfall was generated by a rain distribution system. This system involved five artificial devices, installed on a frame over the pavement. The intensity, raindrop angle, and oscillation frequency of the artificial devices were controlled by a computer program. Two outflow gauges were used to measure the outflow rate according to time of water overflowing from the surface and draining into the subbase of the reinforced pavement system. The rainfall simulation experiment setup and outflow gauges are shown in Figure 11.

## 3. Test Program

After the construction of both the pavement sections, their mechanical characteristics were measured by the PLT and FWD tests, respectively. The hydrologic characteristics of the reinforced section were then evaluated using a rainfall simulation test.

The PLT procedure followed that established by the German standard DIN 18134, with some modifications [14]; it consists of preloading, followed by the first and second loading cycles. The preloading applies a stress of 0.01 MPa to the pavements for at least 30 s to ensure the stability of the loading plate before testing. After finishing this progress, the two LVDTs were set to zero. For the first loading cycle, the load levels were applied in eight stages, according to the manual. After reaching the maximum stress value of 0.5 MPa, the applied stress was reduced to 50% and 25% of the maximum stress value. Finally, it was reduced to a preloading stress value of 0.01 MPa. The second loading cycle was then conducted in the same way as the first, but without the application of maximum stress. The data results, including loads and deformations, were recorded after each loading stage for both cycles.

FWD tests were conducted for the two pavements. The FWD generates a load pulse by dropping a weight, which deforms the pavement surface deflection basins [15]. Numerous computer programs for performing the back-calculation of the layer modulus using FWD deflection basins are widely used; this study used the modulus program [16]. 

Finally, rainfall simulation tests were conducted on the reinforced pavement. During rainfall, the rates of outflow for surface runoff and infiltration through the pavement body were recorded separately by the two flow gauges. The rainfall intensity was selected to be 100 mm/h and was applied for an hour. The outflows were monitored every minute. 

## 4. Results and Discussions

### 4.1. Plate Load Test

From the test results, the values of the elastic modulus for the first and second loading cycles were back-calculated on the basis of the process specified in the manual DIN 18,134 [14]. To describe the stress-strain curve, a second-order polynomial regression analysis was used:

(1) $$s=a_{0}+a_{1}\sigma_{0}+a_{2}\sigma_{0}^{2}$$

where *s* is the settlement of the plate (mm), *σ*_0_ is the normal stress below the plate (MPa), and *a*_0_, *a*_1_, and *a*_2_ are constants. 

Based on the stress-strain curves, the elastic modulus values for the first and second loading cycles were determined according to the manual DIN 18134, which is presented in Equation (2):

(2) $$E_{v}=1.5\times r\times \frac{1}{a_{1}+a_{2}\times \sigma_{0\max}}$$

where *E_v_* is the elastic modulus (MPa), *r* is the radius of the plate (mm), and *σ*_0max_ is the maximum normal stress below the plate.

In this study, the elastic modulus of the pavement at a deformation of 2.5 mm, known as the modulus of the subgrade reaction (*k*_2.5_), is expressed as:

(3) $$k_{2.5}=\frac{\Delta \sigma_{0}}{s}k$$

where ∆*σ*_0_ is the difference between the preloading stress and the stress corresponding to a deformation of 2.5 mm, and *s* is the deformation (2.5 mm).

**Figure 12:**
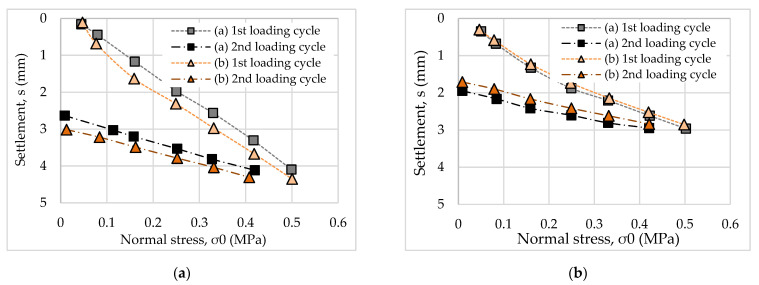
PLT results for two tests (a and b) of the first and second loading cycle: (**a**) unreinforced pavement; (**b**) reinforced pavement.

In this study, each pavement was tested twice via PLTs (a and b). On the basis of the results, the stress-strain curves were established, as shown in Figure 12.

It can be seen that, for the same normal stress level, the settlement of the unreinforced pavement was higher than that of the reinforced pavement. It is evident that the use of geocell composites increased the mechanical characteristics of the porous asphalt pavement. For both pavements, the results of the two tests (a and b) did not vary considerably. Thus, the two pavements were compacted well before testing. 

On the basis of these curves, the elastic modulus of the two pavements was calculated according to Equations (1) and (2). In addition, the modulus of the subgrade reaction according to Equation (3) was analysed. The results are presented in Table 1. 

The results indicate that the elastic modulus of the unreinforced pavement was lower than that of the reinforced pavement. This behaviour is similar to the results presented by Khalaj et al. (2015), who used a cyclic PLT to investigate the effects of the geocell composite on the pavement base layer [10]. The reason could be explained by the fact that the geocell composite distributed the load from the surface of pavement to the underlying layers with a wider area [17]. 

When comparing the results of the two test loading cycles, the elastic modulus of the second loading cycle was approximately two times larger than that of the first. According to Alexiew (2008), the ratio of the elastic modulus during the second to first loading cycle should be approximately two for good compaction [18]. Therefore, it can be concluded that the two pavements in this study were well compacted for the experiments. 

According to the manual ZTV E-StB 09 [19], the results of *E_v_*_2_ of pavement layer should be at least 70 MPa. Considering the results in Table 1, it can be seen that the average result of *E_v_*_2_ was 87.68 MPa. This indicates that the reinforced pavement met the requirement for modulus. 

In this study, the value of modulus of subgrade reaction for the reinforced pavement was higher than 1.5 times of that for the unreinforced pavement. It can be seen that in a small portion of the stress-displacement curve, the reinforced pavement yielded a consistently higher modulus than the unreinforced pavement. According to Pancar and Akpinar [20], the requirement of modulus of subgrade reaction for the pavement reinforced by geocell is 18.33 MPa/m. In this study, the reinforced pavement had the modulus of subgrade reaction as about 150 MPa/m. Therefore, it can be concluded that the reinforced pavement meets the requirement of the modulus of the subgrade reaction. 

### 4.2. Falling Weight Deflectometer Test

**Figure 13:**
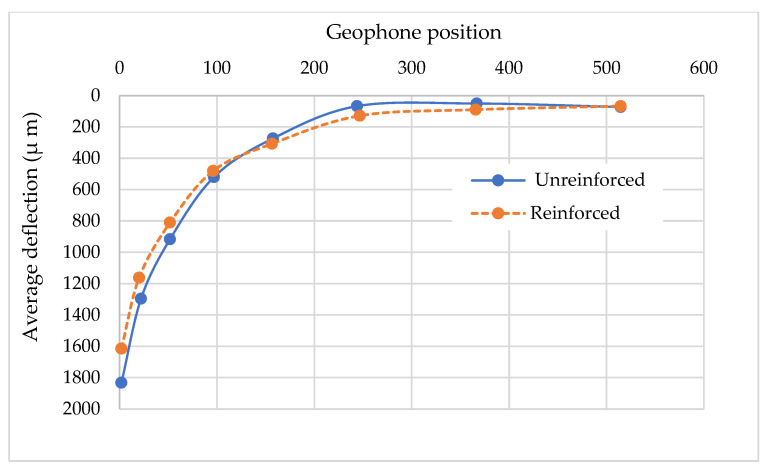
Average of FWD deflection basins layout.

The average deflection data of the two pavements according to the geophone position are presented in Figure 13. It can be seen that the deflection values of the upper layer corresponding to geophone measurements from no. 1 to no. 4 for the reinforced pavement were generally less than those from the unreinforced pavement. It should be noted that the deflection values of the subbase layer corresponding to the geophone measurements from no. 5 to no. 7 for the reinforced pavement were slightly higher than those of the unreinforced one. It could be attributed to the use of aggregate material for the subbase layer. While the open-graded aggregate material with a nominal maximum aggregate size of 45 mm was used for the subbase of the reinforced pavement, one with 10 mm was used for that of the unreinforced pavement. Therefore, the mechanical characteristic of the subbase layer of the reinforced pavement is lower than that of the unreinforced pavement. However, it might not be crucial for the structural adequacy of the reinforced pavement because most of the structural adequacy is supported by the upper layer. 

On the basis of the results in Figure 13, it can be concluded that the geocells enhance the load-bearing capacity of the upper layers, resulting in reduced deflection basins. According to Emersleben and Meyer [21], the use of geocell could make an all-around confinement to the reinforced layer. This could help the applied vertical load to be transferred over a larger area. Therefore, the vertical stresses from vertical load on the reinforced layer are reduced so that the deformation of the reinforced layer could be decreased. 

The layer elastic modulus was then back-calculated for comparison. In the analysis, the pavements were divided into three layers. For the pavement with a geocell composite, the base layer was regarded as a typical base layer, with a thickness of 300 mm. Numerous computer programs for performing the back-calculation of layer modulus using FWD deflection basins are widely used. In this study, the Modulus program 7.0 was used for this purpose [16]. The program requires the seed modulus, range of acceptable modulus, layer thickness, Poisson’s ratio, and FWD loads as inputs. To analyse the modulus results, an advanced segmentation routine was used. Details of the procedure can be found in the manual [16]. The sum of the relative squared error between the predicted and measured FWD deflections was employed as a convergence scheme, defined as follows [22]:

(4) $$\mathrm{minimise}\;\varepsilon^{2}={\sum_{i=1}^{s}\left[\sum_{j=1}^{d}\left(\frac{w_{ij}^{m}-w_{ij}^{c}}{w_{ij}^{m}}\right)\right]}^{2}we_{i}$$

where *ε*^2^ is the squared error, $w_{ij}^{m}$ is the measured deflection, $w_{ij}^{c}$ is the computed deflection, *i* is the sensor number sequence, *j* is the test number sequence, and *we_i_* is the weighting factor of sensor *i*.

The results of the elastic modulus back-calculation in the program are presented in Table 2 and Table 3.

The average absolute error of the back-calculated layer modulus was 17.37% for the unreinforced pavement and 16.69% for the reinforced pavement, which is in accordance with the guideline that the error needs to be less than 20% [16]; hence, this is acceptable. Note that the 7th station (0.214) of the unreinforced pavement and the 9th station (0.216) of the reinforced pavement exhibited the lowest back-calculated modulus value, which was only 50 ksi. These are mainly attributed to the higher FWD deflection in the first sensor for the two pavements.

To quantify the effectiveness of the geocell, layer elastic analysis was conducted to determine the equivalent base thickness of the unreinforced pavement section. As presented in Table 4, when the base layer thickness increased to 600 mm, the predicted FWD deflection basins were similar to that of the geocell-applied section by showing the least error. 

In other words, the use of a geocell composite reduces the thickness of the base layer, which could save material and construction costs. This result supports the conclusions of previous studies [11,16]. 

### 4.3. Retention of Water Volume

To examine the hydrologic characteristics of the reinforced pavement, rainfall simulation tests were conducted. The percentage of water retention was used to evaluate the hydrologic characteristics, expressed as following:

(5) $$\mathrm{Retention}\;\left(\%\right)=\frac{\mathrm{Inflow}-\mathrm{Outflow}}{\mathrm{Inflow}}\times 100\%$$

**Figure 14:**
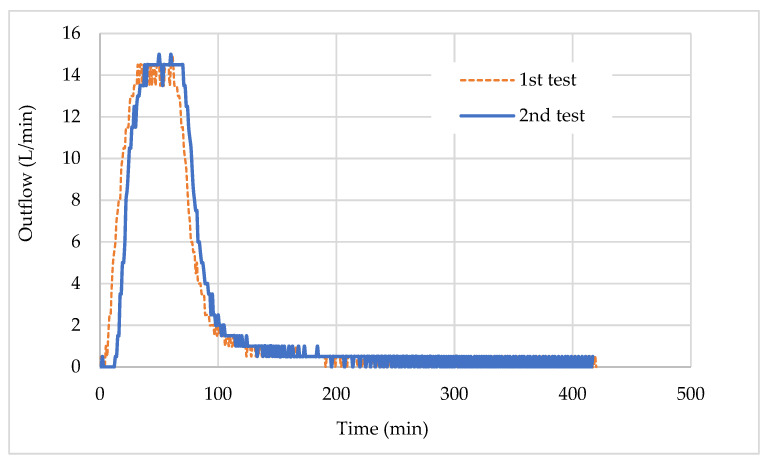
Rate of water outflow with respect to time.

Under rainfall, water infiltrates through the pavement system and starts to drain out. The surface runoff and outflow water were recorded continuously. The results show that there is no surface ponding for both trials; the reinforced porous asphalt pavements worked well for handling surface runoff. The time histories of water outflows are presented in Figure 14. 

The results showed that the water started to drain out of the pavement at 13 min and 5 min for the first and second tests, respectively. Based on Figure 14, the duration of the maximum infiltration rate (peak rate) of the two tests were not significantly different, i.e., it took 32 min and 38 min for the first and second tests, respectively.

The volume of water was monitored for 48 hours after completion of each test. The final water volume of surface runoff, outflow, and retention normalized by inflow are listed in Table 5. From the results, it can be seen that there was no surface runoff for the reinforced porous asphalt pavement. It can be concluded that the reinforced porous asphalt pavement effectively handled stormwater. It is however noted that, according to Fassman and Blackbourn [23], the time for surface ponding of permeable pavement system was about 2.4 h and strongly depended on the rainfall intensity. 

## 5. Conclusions

This study presents an investigation of the mechanical and hydrologic characteristics of the porous asphalt pavement, with and without geocell composites, using PLT, FWD, and rainfall simulation tests. The conclusions drawn are as follows. 

The elastic modulus of the unreinforced pavement is lower than that of the reinforced pavement. A comparison of the results of the two loading cycles showed that the elastic modulus of the second loading cycle was approximately two times larger than that of the first. The elastic modulus back-calculated from the FWD data showed that the layer modulus of the reinforced pavement was higher than that of the unreinforced pavement. 

The equivalent thickness of the reinforced pavement to the unreinforced pavement was determined. Based on the results, the unreinforced pavement with a base layer thickness of 600 mm provided the same predicted elastic modulus values as the reinforced pavement. It was concluded that the designers can reduce the pavement thickness when using a geocell composite for the base layer. 

There was no surface ponding in the rainfall simulation test after providing the rainfall with an intensity of 100 mm/h for an hour. This indicates that the reinforced porous pavement effectively handled surface runoff.

In future, for wide implementation of porous asphalt and reinforcement, more studies are needed to evaluate the mechanical and hydrologic characteristics of porous asphalt pavement with considering the effect of freeze/thaw cycle and traffic load.

## Figures and Tables

**Table 1:** PLT results for the two pavements.

Pavement	Loading Cycle #	PLT Results				
		*E_v_*_1_ (MPa)	*E_v_*_2_ (MPa)	*E_v__2_/E_v_*_1_	*k*_2.5_ (MPa/m)	Settlement at 0.5 MPa (mm)
Unreinforced	1	26.26	64.31	2.45	119.80	4.13
	2	24.44	66.92	2.74	100.86	4.38
	Average	25.35	65.61	2.59	110.33	4.25
Reinforced	1	38.03	93.29	2.45	145.46	2.97
	2	38.39	82.07	2.14	153.17	2.90
	Average	38.21	87.68	2.30	149.32	2.94

**Table 2:** Elastic modulus back-calculation results for the unreinforced pavement in the program.

Station	Load (lbs)	Surface	Base	Subbase	Subgrade	Layer	Absolute Err/Sens (%)
		2	12	20	26.31	Thickness (in)	
		0.35	0.35	0.35	0.35	Poisson Ratio	
		Calculated Modulus Values (ksi)				Depth to Bedrock	
		*E*_1_	*E*_2_	*E*_3_	*E*_4_		
0.041	9.984	223.1	11	10	1.8	59.8	15.42
0.042	9.842	231.9	11	10	1.8	61.6	15.04
0.043	9.862	245.6	11	10	1.8	61.8	14.89
0.104	9.802	145.6	11	10	2.3	57.7	18.56
0.105	9.979	255.3	11	10	2.3	55.6	16.83
0.106	10.127	295.0	11	10	2.3	55.1	15.84
0.214	9.827	50.0	11	10	2.3	57.7	21.06
0.215	10.16	122.6	11	10	2.3	57.5	20.05
0.216	10.054	152.4	11	10	2.2	56.2	18.67
Average		191.3	11	10	2.1	58.0	17.37

**Table 3:** Elastic modulus back-calculation results for the reinforced pavement in the program.

Station	Load (lbs)	Surface	Base	Subbase	Subgrade	Layer	Absolute Err/Sens (%)
		2	12	20	32.87	Thickness (in)	
		0.35	0.35	0.35	0.35	Poisson Ratio	
		Calculated Modulus Values (ksi)				Depth to Bedrock	
		*E*_1_	*E*_2_	*E*_3_	*E*_4_		
0.041	10.142	309.6	11	10	2.9	65.7	15.9
0.042	10.254	421.9	11	10	2.9	68.0	14.92
0.043	10.239	472.1	11	10	2.9	69.4	14.56
0.104	10.045	208.2	11	10	3.1	67.3	17.65
0.105	10.210	300.1	11	10	3.2	67.5	16.35
0.106	10.272	353.5	11	10	3.2	68.0	15.65
0.214	10.149	252.2	11	10	3.3	64.5	18.09
0.215	10.274	335.9	11	10.3	3.4	65.5	16.66
0.216	10.252	50.0	16	10.3	3.4	66.2	20.41
Average		300.4	11.6	10.1	3.2	66.9	16.69

**Table 4:** Back-calculated elastic modulus results for the unreinforced pavement program according to different thicknesses.

Result	Thickness (mm)	Geophone Number								Ave. Error ^1^ (μm)
		0	1	2	3	4	5	6	7	
Reinforced pavement	300	1658	1192	824	496	314	136	87	76	-
Analysis for unreinforced pavement	300	1882	1193	830	531	375	223	140	96	60.8
	400	1811	1134	783	503	361	220	139	94	32.8
	500	1767	1096	752	484	352	206	145	100	14.9
	600	1736	1070	730	468	342	222	149	104	4.8

^1^ Average error between FWD deflection basins of reinforced and unreinforced pavements.

**Table 5:** Results for rainfall simulation test for reinforced porous asphalt pavement.

Test no.	Normalized Water Volume (%)		
	Surface Runoff	Outflow	Retention
1	0	80.3	19.7
2	0	80.2	19.8
